# Small molecule inhibitors from organoid‐based drug screen induce concurrent apoptosis and gasdermin E‐dependent pyroptosis in colorectal cancer

**DOI:** 10.1002/ctm2.812

**Published:** 2022-04-12

**Authors:** Yuchen Zhang, Zhuoqing Xu, Wenqing Feng, Han Gao, Zifeng Xu, Yiming Miao, Wenchang Li, Fangqian Chen, Zeping Lv, Jianting Huo, Abudumaimaitijiang Tuersun, Wangyi Liu, Yaping Zong, Xiaohui Shen, Jingkun Zhao, Aiguo Lu

**Affiliations:** ^1^ Department of General Surgery Ruijin Hospital Shanghai Jiaotong University School of Medicine Shanghai China; ^2^ Shanghai Minimally Invasive Surgery Center Ruijin Hospital Shanghai Jiaotong University School of Medicine Shanghai China; ^3^ Department of Radiology Ruijin Hospital Shanghai Jiaotong University School of Medicine Shanghai China

Dear Editor,

The existing preclinical drug models to screen cancer drug candidates are inefficient in achieving satisfactory results for clinical applications, and chemotherapy resistance remains a major problem in colorectal cancer. To search for effective therapeutic drugs, we innovatively employed a cancer tissue‐originated spheroids (CTOS)‐based screening method to identify personalised treatments and predict drug responses for neoadjuvant therapy.

Testing of new drug candidates is predominantly performed in cancer cell lines, but these cell lines do not retain the characteristics of the original tumours they were derived from, in part due to the numerous passages.^1^ To overcome the low efficacy of therapeutic screening and take inter‐patient variations into account, we established colorectal cancer patient‐derived organoids through CTOS methods.^2^, ^3^ After tissue dissociation, the cell aggregates with diameters of 40–100 μm were collected for further culture (Figure 1A). CTOS appeared as irregular fragments immediately after filtration and then formed transparent and smooth spheres after culture (Figure S1A). Inter‐ and intratumour heterogeneity was observed in these CTOS, each retaining different differentiation degrees of the original adenocarcinomas with poorly differentiated lesions appearing as solid growths and well‐differentiated ones as crypt‐like (Figure S1B,C). The mutations of *KRAS*, *BRAF*, *TP53* and *PIK3CA* matched well between CTOS and the original tumours (Figure 1B, Table S4). Histological characteristics, epithelial marker expression (EpCAM), proliferation level (Ki67) and mucin‐producing ability (MUC2) were confirmed to have strong concordance with the respective tumours (Figure 1C,D). The extracted cell aggregates consisted of almost pure tumour cells with few immune or vascular endothelial cells (Figures 1E and S1D,E). Since cancer stem cells have an intrinsic mechanisms of drug resistance,^4^ the expression of the stemness markers CD133, CD166 (Figure 1F,G) and Lgr5 (Figure S1F) was evaluated, and they were slightly elevated during culture.

**FIGURE 1:**
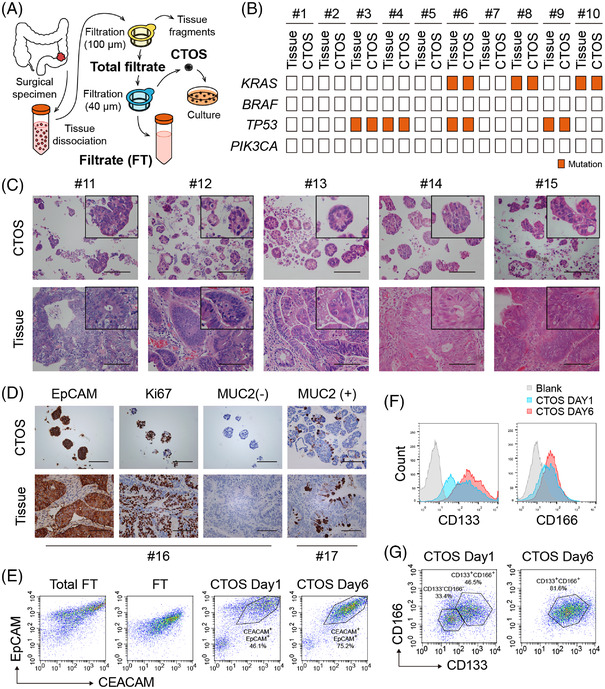
Cancer tissue‐originated spheroids (CTOS) phenotypically represent original colorectal cancer tumours. (A) Schematic diagram of colorectal cancer (CRC) CTOS preparation. In brief, surgical specimens of CRC were digested mechanically and enzymatically. After filtration of tissue dissociation, cell aggregates were obtained over the 40 μm filter and cultured for further assays. (B) Mutation profiles of 10 pairs of CTOS and primary tumours in *KRAS*, *BRAF*, *TP53* and *PIK3CA* genes. (C) Haematoxylin and eosin (H&E) staining of CTOS and corresponding tumour tissues. (D) Immunohistochemistry (IHC) staining of CTOS and corresponding tumour tissues marked with EpCAM, Ki67 and MUC2. (E) Flow cytometry analysis of total filtrate, filtrate (FT) and CTOS #14 cultured on days 1 and 6, stained by epithelial markers CEACAM and EpCAM. (F and G) Flow cytometry of CTOS #14 cultured for 1 and 6 days, stained by stem cell markers CD133 and CD166. Scale bar = 200 μm

It remained to be clarified, however, the availability of our CTOS model in the screening of a drug candidate, the detection of drug‐induced cell death and its role in the immune response. Initially, a panel of 60 drugs (Table S2) was tested in our CTOS‐based screening assay (Figure 2A). The after‐treatment morphological changes of the spheroids directly indicated the drug effects. The signs of damaged cells included low transparency, disorganised shape with an irregular surface and disaggregation into single cells (Figure S1C). Our method of cell viability measurement proved to have similar inspection efficiency as evaluations by adenosine triphosphate (ATP) levels (Figures S1G,H). We identified four hit inhibitors, obatoclax mesylate (OM), BI 2536 (BI), (S)‐(+)‐camptothecin (CPT) and bortezomib (BTZ), showing marked growth suppression of the CTOS (Figure 2B–E). All agents demonstrated effective growth inhibition in a dose‐ and time‐dependent manner and marked treatment‐induced apoptosis (Figure S2). However, apoptotic cancer cells incapable of killing themselves tend to gain other mutations, leading to genetic instability and diversity of malignant cells.^5^ If different types of cell death operate synergistically, the toxicity of the agent may be reduced.

**FIGURE 2:**
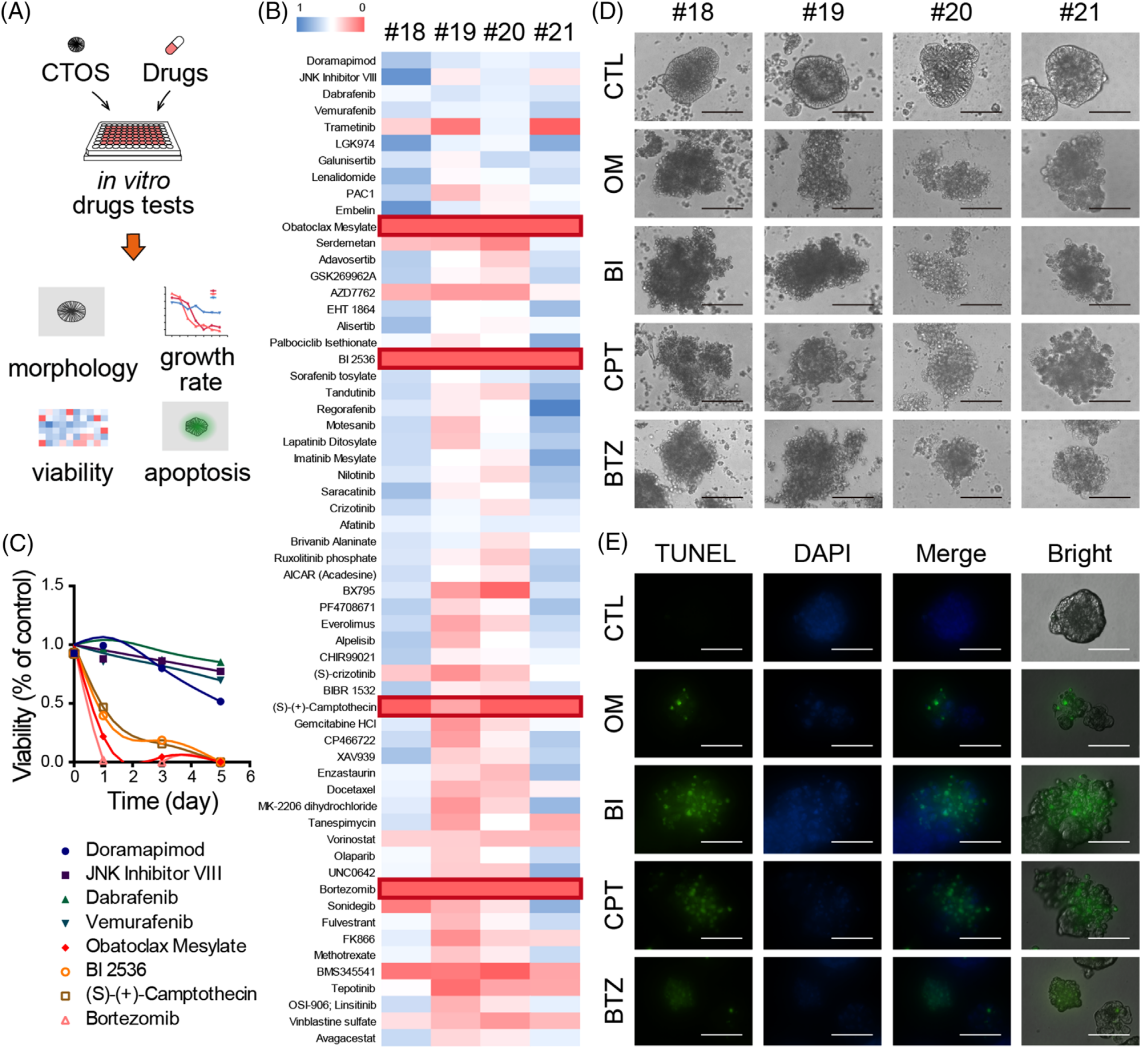
Several small molecule inhibitors suppress colorectal cancer tissue‐originated spheroids (CTOS) growth by apoptosis during drug screening. (A) Sketch map of CTOS‐based drug screening assay. (B) Heatmap of viability of CTOS derived from four colorectal cancer (CRC) patients after treatment with 60 small molecule inhibitors for 5 days. (C) Time‐viability curves of four sensitive and four non‐sensitive drugs selected from the screen using CTOS #18. (D and E) Representative images and TUNEL assay of CRC CTOS treated with obatoclax mesylate (OM), BI 2536 (BI), (S)‐(+)‐camptothecin (CPT) and bortezomib (BTZ). The CTOS used in TUNEL assay is CTOS #13. Scale bar = 200 μm

Interestingly, when examining caspase‐3 activity, we noticed that drug‐damaged cells showed balloon‐like bubbles distinct from classic apoptotic blebbing (Figure S2D). This was reminiscent of the typical pyroptotic morphology. We observed their appearance in culture, and the colorectal cancer cells, especially the suspension ones, were swelled and exhibited large bubbles of the plasma membrane after treatment (Figure 3A). We verified the existence of gasdermin E (GSDME) expression in SW1463 and SW620 cells (Figure S3A). The release of lactate dehydrogenase (LDH) and high mobility group proteins B1 (HMGB1) in culture medium was elevated in cells treated with the four focused drugs (Figure 3B,C), indicating cell membrane rupture and leakage. Cells undergoing necroptosis have a similar swelling phenotype and change in membrane permeability.^6^ To distinguish which pathway OM, BI, CPT and BTZ induced cell death through, we examined relative protein levels by western blot. Cleavage of both caspase‐3 and poly (ADP‐ribose) polymerase 1 (PARP1, markers of apoptosis) was observed in BI‐ and CPT‐treated colorectal cancer cells, and GSDME, though not gasdermin D (GSDMD), was cleaved to generate N‐terminal fragments (markers of pyroptosis, Figures 3D and S3E). No changes in mixed lineage kinase domain‐like protein (MLKL) phosphorylation (a marker of necroptosis) appeared after OM, BI or CPT exposure (Figure 3E), while BTZ‐treated cells underwent necroptosis instead of pyroptosis (Figure 3D,E). Moreover, when treated, the peripheral cells of CTOS first exhibited large bubbles of the membrane, and single cells dissociated from the spheroids (Figure 3F). We then employed siRNA to verify whether BI‐ and CPT‐induced GSDME‐mediated pyroptosis was dependent on caspase‐3.^7^ After GSMDE knockdown, the pyroptotic phenotype was diminished without an influence of caspase‐3 activation (Figure 3G,J). After the knockdown of caspase‐3, the expression of both cleaved caspase‐3 and the N‐terminal fragments of GSDME were downregulated (Figure 3J), suggesting that the activation of caspase‐3 regulates the cleavage of GSDME in BI‐ and CPT‐induced pyroptosis of colorectal cancer cells. The release of LDH and HMGB1 was markedly decreased after the knockdown of GSDME or caspase‐3 (Figure 3H,I). The above results were also confirmed with a caspase‐3 inhibitor, Z‐DEVD‐FMK (DEVD) (Figure S3G–K), implying the pyroptosis was secondary to the activation of caspase‐3.

**FIGURE 3:**
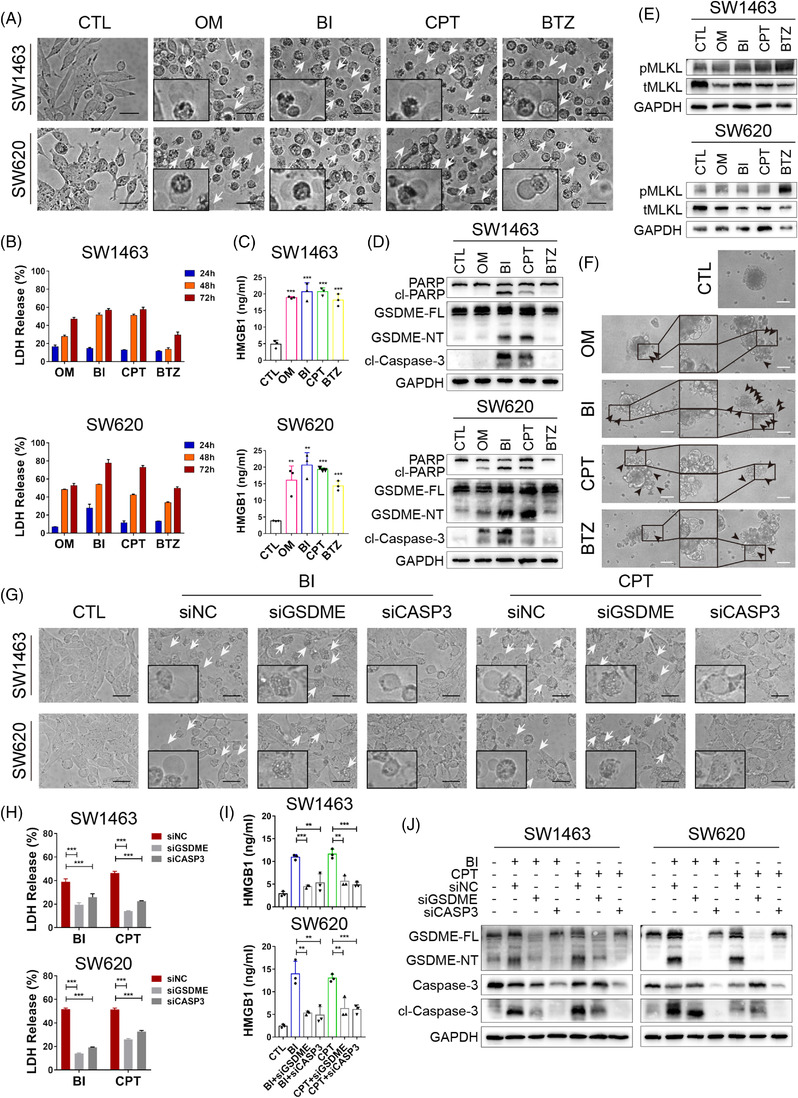
BI 2536 (BI) and (S)‐(+)‐camptothecin (CPT) induce gasdermin E (GSDME)‐dependent pyroptosis accompanied by apoptosis. (A) Representative images of SW1463 and SW620 cells treated with DMSO, obatoclax mesylate (OM), BI, CPT and bortezomib (BTZ) for 72 h. Pyroptotic cell morphology is pinpointed by arrows. (B) LDH and (C) high mobility group proteins B1 (HMGB1) release from SW1463 and SW620 cells treated with indicated inhibitors. Each column represents the mean value of three biological replicates, and error bars indicate standard deviation (SD). (D and E) Immunoblotting analysis of the indicated proteins extracted from inhibitor‐treated cells. BI‐ and CPT‐induced cleavage of PARP, GSDME and caspase‐3, and BTZ‐induced phosphorylation of MLKL is demonstrated. (F) Representative images of colorectal cancer (CRC) organoids treated with indicated inhibitors, 1 μM for OM, 0.5 μM for BI, 0.1 μM for CPT and 0.01 μM for BTZ. (G) Bright‐field microscopy images of indicated inhibitor‐treated SW1463 and SW620 cells. Altered morphology was shown when GSMDE or caspase‐3 were knockdown. (H) LDH and (I) HMGB1 release of CRC cells treated with BI and CPT. The level of LDH decreased when GSMDE or caspase‐3 were knockdown. (J) Immunoblotting analysis of the indicated proteins extracted from inhibitor‐treated cells. Scale bar = 200 μm. CTL, control; cl‐PARP, cleaved‐PARP; GSDME‐FL, full‐length GSDME; GSDME‐NT, GSDME N‐terminal domain; cl‐Caspase‐3, cleaved‐Caspase‐3; pMLKL, phosphorylated MLKL; tMLKL, total MLKL; siNC, si‐negative control; siGSDME, si‐gasdermin E; siCASP3, si‐caspase‐3

In vivo drug responses were validated with the following strategy. BLAB/c‐nude and BLAB/c mice bearing subcutaneous tumours were treated with dimethyl sulfoxide (DMSO), OM, BI, CPT or BTZ via intraperitoneal and intratumoural injection every other day four times (Figure 4A). After the treatments, the growth rates of the tumours in either immunocompetent mice or immunodeficient mice were robustly inhibited compared with the vehicle control values (Figure 4B,C). In addition, no significant toxicity to the liver or kidney was visible, according to their morphology (Figure S4A,B). We also confirmed the drug efficacy in the patient‐derived xenograft model (Figure S4C–E). Moreover, we surprisingly found that mice with an intact immune system exhibited better therapeutic effectiveness than nude mice (Figure 4B). Thus, we speculated that these small molecule drugs altered the immune response. As depicted in Figure 4E,F, CD4^+^ and CD8^+^ T cells were clustered in the tumour sites after treatment.

**FIGURE 4:**
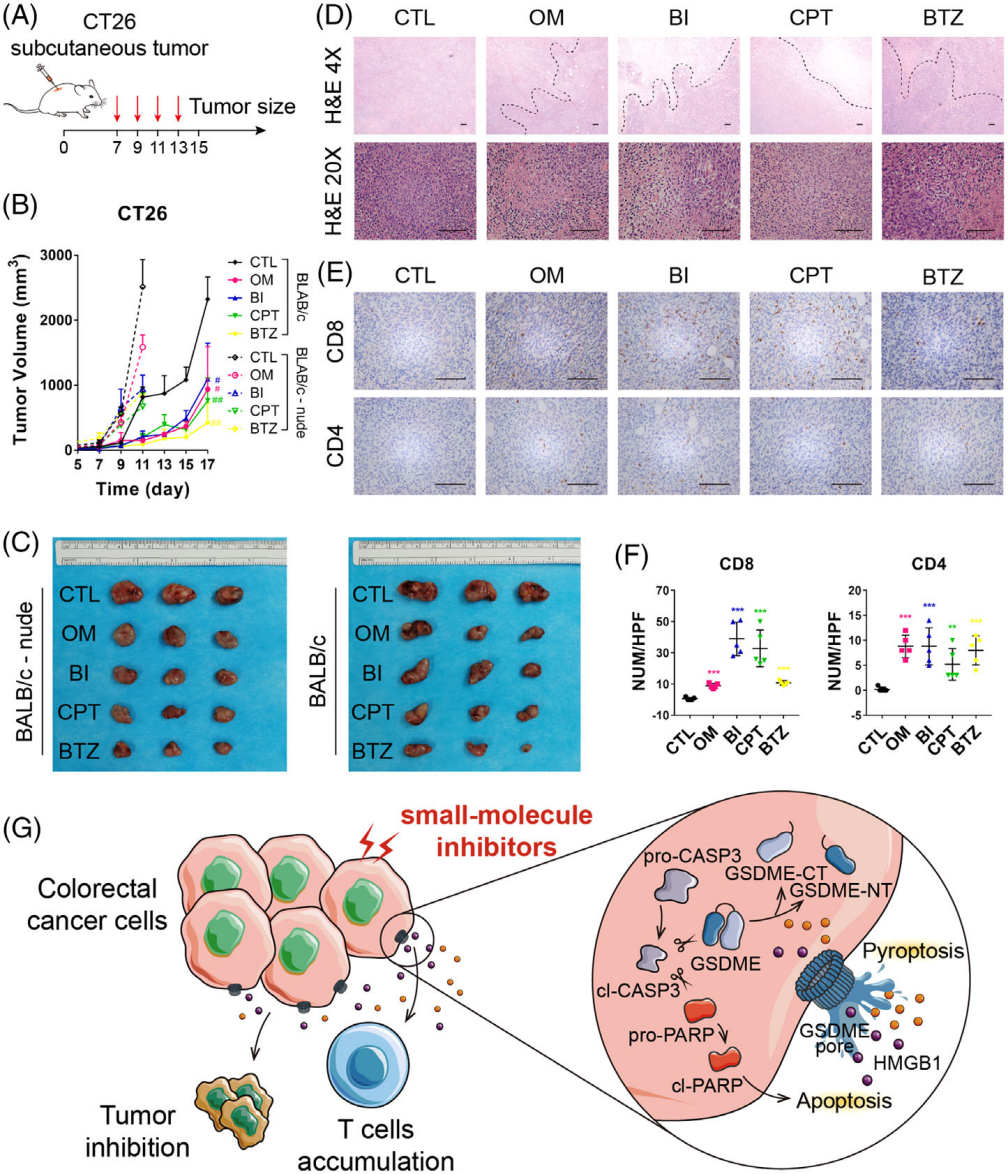
Several small molecule inhibitors suppress colorectal cancer tumour growth and accumulate CD8^+^ T cells in vivo. (A) The strategy of in vivo drug treatment. (B) Tumour growth curves and (C) images of xenograft tumours for DMSO, obatoclax mesylate (OM), BI 2536 (BI), (S)‐(+)‐camptothecin (CPT) and bortezomib (BTZ) treatment among BALB/c‐nude and BALB/c mice. (D) Haematoxylin and eosin (H&E) staining of drug‐treated subcutaneous tumours in BALB/c mice. The dotted lines indicated large areas of cell death in drug‐treated tumours, but no necrosis was found in the control one. (E) Immunohistochemistry (IHC) staining of CD8 and CD4 in inhibitor‐treated tumours in BALB/c mice. (F) The number of positive cells in each high‐power field (HPF). (G) A schematic representation of the proposed mechanism for gasdermin E (GSDME)‐dependent pyroptosis induced by small molecule inhibitors in colorectal cancer (CRC). Scale bar = 200 μm

In conclusion, using our strategy of CTOS‐based drug screening, four drug candidates for colorectal cancer treatment were found. Among them, two small molecule inhibitors, BI and CPT, were further verified to induce GSDME‐mediated pyroptosis concurrent with caspase‐3‐dependent apoptosis, and they showed both in vitro and in vivo antitumoural activity (Figure 4G). Pyroptosis induced by BI and CPT is an unrecognised mechanism that may feasibly enhance the efficacy of immunotherapy.

## CONFLICT OF INTEREST

The authors have declared that no conflicts of interest exists.

## Supporting information

Supporting Information
