# Supplementary material for: Small molecule inhibitors from organoid‐based drug screen induce concurrent apoptosis and gasdermin E‐dependent pyroptosis in colorectal cancer
Source: Clin Transl Med. 2022 Apr 12;12(4):e812. doi: 10.1002/ctm2.812 (PMC9005931; doi:10.1002/ctm2.812)
Supplement: Supplementary file 1 — Supporting Information [file CTM2-12-e812-s001.docx]

**Supporting Information**

**Supplementary Materials and Methods**

**Cell culture and reagents**

Human CRC cell lines SW1463 and SW620 cells were purchased from the American Type Culture Collection (ATCC). Mouse CRC cell line CT26 was purchased from Shanghai Institutes for Biological Sciences, Chinese Academy of Sciences. All cells were cultured in RPMI 1640 medium (Meilunbio) supplemented with 10% fetal bovine serum (FBS; Gibco) and 1% penicillin/streptomycin (Gibco) in a humidified incubator at 37 ℃ in 5% CO2. Small molecule inhibitors, including obatoclax mesylate (OM), BI 2536 (BI), (S)-(+)-camptothecin (CPT), bortezomib (BTZ), Z-DEVD-FMK (DEVD) and a library of 130 compounds (TargetMol) either approved by the FDA or used in human clinical trials, were dissolved in dimethyl sulfoxide (DMSO) at a storage concentration of 10 mM.

**Patient samples**

All patients went through colonoscopy and their endoscopic biopsies were confirmed to be malignant pathologically. Tumor specimens (Table S1) were acquired from patients with CRC who underwent surgical resection at Shanghai Minimally Invasive Surgery Center, Ruijin Hospital. None of the patients received any treatment prior to the surgery. For each case, available tissue was used for CTOS preparation, genomic and histological analysis. This study was approved by Ruijin Hospital Ethics Committee and written informed consent was obtained from all patients involved.

**CTOS preparation and chemosensitivity assay**

Fresh tumor samples were collected and preserved in phosphate-buffered saline (PBS) containing ciprofloxacin. Preparation and culture of the CTOS were performed according to the established protocol [8]. Briefly, surgical samples were mechanically minced and digested in Dulbecco’s modified Eagle’s medium (DMEM)/F12 medium (Meilunbio) containing collagenase Ⅳ (Sigma-Aldrich) for 45–60 minutes at 37 ℃ with continuous stirring. The digested solution was filtered through cell strainers. Fragments between 100- and 40-μm were collected and cultured for 24–48 hours in OriCell® hESC Medium (Cyagen Biosciences) without serum to form CTOS.

The primary CTOS was then seeded in 96-well plates (Corning Costar) with an amount of 50–150 CTOS to each well. Sixty drugs (Table S2) were select from our compound library and dispensed into 96-well plates at a final concentration of 3μM. Control wells (DMSO-treated) were placed in each plate. After the addition of drugs, the CTOS were cultured for another five days. The alive CTOS after treatment were coated with debris and retained bright and smooth spheres in the core, counted at day 1 and 5. The ratio of numbers at day 1 and 5 was recorded as the relative viability of CTOS treated after each compound.

**Detection of gene mutations**

The DNA of tumor tissues and CTOS was extracted with Magnetic Bead Method Tissue DNA Extraction Kit (DE0596B, Emerther) according to the manufacturer’s instructions. Polymerase chain reaction (PCR) was used to amplify exon 2 of the *KRAS* gene, exon 7 and 8 of the *TP53* gene, exon 15 of the *BRAF* gene, which potentially contained the mutation encoding BRAF^V600E^, and the sequence containing the 1633^rd^ base of *PIK3CA*. The primers were listed in Table S3. After the purification of the target DNA fragments, Sanger sequencing was used to detect the mutations. The reaction was performed as follows: 94°C for 1 minutes (1 cycle), 94°C for 20 seconds, 50°C for 10 seconds and 60°C for 4 minutes (28 cycles) and the reaction was then held at 4°C. The purified products of the cycle sequencing were analyzed on the 3730xl DNA Analyzer (Applied Biosystems). Sequencing Analysis 5.2.0 software and Sequencher 5.1 package were used to assemble the sequences and align with the reference genomes.

**Hematoxylin-eosin (H&E) and immunohistochemistry (IHC) staining**

For H&E staining, tissue samples were fixed in 4% paraformaldehyde, washed with PBS and transferred to 70% ethanol. The samples were then embedded in paraffin, sectioned and stained with H&E. For IHC staining, paraffin-embedded tissues were deparaffinized in xylene, passed through graded alcohols and the antigen was retrieved with citrate buffer in a steam pressure cooker. The samples were then incubated with anti-EpCAM (ab223582, Abcam), anti-Ki67 (ab15580, Abcam), anti-MUC2 (ab272692, Abcam), anti-CD4 (ab183685, Abcam) and anti-CD8 (ab217344, Abcam), washed in PBS, and incubated with horseradish peroxidase-conjugated secondary antibody. Slides were counterstained with hematoxylin, dehydrated in graded alcohol and xylene, and coverslipped with mounting solution. The number of positive cells were counted in each high-power field (HPF) by two independent pathologists.

**Flow cytometry and apoptosis assay**

For CTOS evaluation, the cells were dissociated to single cells using Accutase (Sigma-Aldrich). The tumor cells were washed with fluorescence activated cell sorting (FACS) buffer [PBS containing 5% bovine serum antigen (BSA)], stained with the following antibodies: human epithelial cell adhesion molecule (EpCAM) allophycocyanin (APC; 347200, BD Bioscience), human CEACAM peridinin chlorophyll protein (PerCP)-Cy5.5 (342311, BioLegend), human CD31 phycoerythrin (PE; 560983, BD Bioscience), human CD45 fluorescein isothiocyanate (FITC; 340664, BD Bioscience), human CD133 APC (394009, BioLegend), human CD166 PE (343903, BioLegend), and human Lgr5 APC (130-100-854, Miltenyi Biotec) for 30 minutes at 4 ℃ in the dark, washed with FACS buffer, and finally detected with flow cytometer. All the data was analyzed with FlowJo version 10 (BD Biosciences).

For the apoptosis assay, after treatment with drugs or the DMSO control, the cells were stained with Annexin-Ⅴ-APC (550474, BD Biosciences) and propidium iodide (PI; 556463, BD Biosciences) according to the manufacturer’s protocol and assessed with a flow cytometer (BD Biosciences). Annexin-Ⅴ^+^ PI^-^ cells were classified as early apoptotic cells, and Annexin-Ⅴ^+^ PI^+^ cells were classified as late apoptotic cells.

**Cell viability assay**

Cell survival rates were estimated by the Cell Counting Kit (CCK)-8 assay (Beyotime Biotec). Approximately 8,000 cells were seeded in 96-well plates with 100 μl of medium in each well. After 24 hours, different concentrations of small molecule inhibitors were added for a three-day treatment. Each well was incubated with 10 μl CCK-8 solution for two hours at 37 ℃ in the dark, and the absorbance at 450 nm was measured by a microplate spectrophotometer (Tecan).

**Immunofluorescence for caspase-3 and TUNEL assays**

For caspase-3 detection, the cells were seeded in φ8 mm cover glasses for growth followed by drug treatments for three days. Visualized apoptotic cells were labelled using GreenNuc™ Caspase-3 Assay Kit for Live Cells (Beyotime Biotec) following the manufacturer’s protocol. GreenNuc™ Caspase-3 Substrate contains the recognition sequence of caspase 3, which can be recognized and cleaved in apoptotic cells, thereby releasing activated DNA green fluorescent dye molecules, and detecting the enzymatic activity of caspase-3. For the TUNEL assay, the organoids were treated with small molecule inhibitors and labelled with FITC using the One Step TUNEL Apoptosis Assay Kit (Meilunbio) according to the manufacturer’s recommendations. The nucleus was stained with 4′,6-diamidino-2-phenylindole (DAPI; Beyotime Biotec). Immunofluorescent staining was observed using a fluorescent microscope (Olympus).

**LDH and HMGB1 release assay**

The CRC cell lines were pretreated with the respective drugs for three days. The LDH release was measured with the LDH Release Assay Kit (Beyotime Biotec) according to the manufacturer’s instructions. In brief, 120 μl of supernatant was transferred to a 96-well plate, and the response mixture was added and incubated in the dark for 30 minutes at room temperature. The absorbance value at 490 nm was then measured, referenced by 1000 nm. The LDH release rate was calculated by the formula: (the absorbance of the treated sample - the absorbance of the vehicle treated control) / (the absorbance of the maximum enzyme activity of the cell - the absorbance of the vehicle treated control) × 100%. HMGB1 levels in the supernatant medium of the CRC cells were determined by an enzyme linked immunosorbent assay (ELISA). HMGB1 (Human) Matched Antibody Pair was purchased from Abnova (H00003146-AP41, Taiwan, China) and the human HMGB1 ELISA kit was purchased from Senxiong BioTech (SX01187, Shanghai, China).

**Western blot analysis**

Cells were lysed in RIPA buffer (Solarbio) containing phenylmethylsulfonyl fluoride (PMSF; Sigma-Aldrich) protease inhibitor, and the protein concentration was measured with the Pierce™ BCA Protein Assay Kit (ThermoFisher Scientific). Protein extracts (20 μg) were subjected to sodium dodecyl sulfate polyacrylamide gel electrophoresis (SDS-PAGE), then transferred onto polyvinyl difluoride (PVDF) membranes (Millipore) and blocked with 5% BSA for one hour at room temperature. The membranes were incubated with primary antibodies targeting PARP (ab32138, Abcam), GSDME (A7432, ABclonal), MLKL (A19685, ABclonal), p-MLKL-T357/S358/S360 (AP0949, ABclonal), caspase-3 (ab32351, Abcam), GSDMD (39754, Cell Signaling Technology; 1:1000 dilution) and GAPDH (60004-1-Ig, Proteintech, 1:5000 dilution) at 4 ℃ overnight. After washing three times with Tris Buffered Saline with Tween (TBST) buffer, the membranes were incubated with horseradish peroxidase-conjugated secondary antibodies at room temperature for one hour. Western horseradish peroxidase (HRP) Substrate (Millipore) was added to visualize protein bands.

**siRNA knockdown**

Cells were transiently transfected with specific siRNA (Genomeditech, China) using Lipofectamine™ 2000 (Invitrogen) according to the manufacturer’s instructions. The targeting site sequences were as follows: si-GSDME: GCAUGAUGAAUGACCUGACUUTT; si-caspase-3: CCGAAAGGTGGCAACAGAATT. Western blot was used to confirm the downregulation efficiency of siRNA. After transfection, the small molecule drugs were added and cultured for additional three days before other analyses.

**Cell-derived subcutaneous implantation model**

All animal studies were approved by the Biomedical Ethics Committee of Ruijin Hospital. Tumor cells (3×10^6^) in the logarithmic growth phase were subcutaneously injected into the flanks of 4-week-old male BALB/c or BALB/c nude mice. One week after injection, the mice were randomly divided into five groups of three, and respectively treated with a vehicle control, OM (20 mg/kg), BI (10 mg/kg), CPT (10 mg/kg) or BTZ (1 mg/kg) *via* intraperitoneal and intratumor injection. When four doses of drugs were injected or the diameter of tumors reached twenty-five millimeters, mice were sacrificed. Tumor volumes were calculated as length × width^2^ × 0.5.

**Immunofluorescent staining**

CTOS were fixed with 4% paraformaldehyde, washed with PBS and permeabilized with 0.1% Triton X-100 at room temperature for 5 minutes. After washing with PBS, spheroids were blocked with 5% BSA for 45 minutes. Human cytokeratin 20 (CK20) antibody (ab76126, Abcam) and human Lgr5 antibody (ARH4134, Antibody Revolution) were used to incubate spheroids overnight at 4 ℃. Alexa Fluor® 555 secondary antibody (ab150078, Abcam) was used to incubate spheroids for 45 minutes. DAPI (Beyotime Biotec) was used to counterstain the nucleus. Immunofluorescent staining was observed using a fluorescent microscope (Olympus).

**Cell viability assay based on ATP levels**

The ATP levels which represent the viability of CTOS were detected by CellTiter-Lumi™ Steady Luminescent Cell Viability Assay Kit (Beyotime Biotec) according to the manufacturer’s protocol. ATP can be quantified by measuring the intensity of chemiluminescence through the luciferin luminescence reaction catalyzed by ATP-dependent luciferase. Since the ATP content, as a proportion of the chemiluminescence intensity, can well reflect the number of living cells, the cell viability can be calculated by the chemiluminescence intensity.

**Patient-derived tumor xenograft (PDX) model**

Tumor specimens were minced into approximately 50 mm^2^, and transplanted subcutaneously into the flanks of 4-week-old male BALB/c nude mice. The developed xenograft was divided into 20 equal parts and passaged to nude mice. After two weeks, the mice were randomly divided into five groups, and respectively treated with a vehicle control, OM (20 mg/kg), BI (10 mg/kg), CPT (10 mg/kg) or BTZ (1 mg/kg) via intraperitoneal and intratumor injection. Tumor volumes were measured as described above. The inoculation of the tumor pieces was performed under anesthesia.

**Statistical analysis**

Statistical analysis was performed with GraphPad Prism 7.0 (GraphPad Software). The statistical significance between two groups was analyzed by the Student’s *t* test. All tests were two-tailed, and *P*-values < 0.05 were considered significant (**P* < 0.05, ***P* < 0.01, and ****P* < 0.001). Correlations between variables were determined by Spearman’s correlation coefficient. The predictive value of number ratio for organoid viability was determined by a receiver operating characteristic (ROC) curve. Relative protein expression was analyzed by ImageJ software.

**Supplementary Figures**


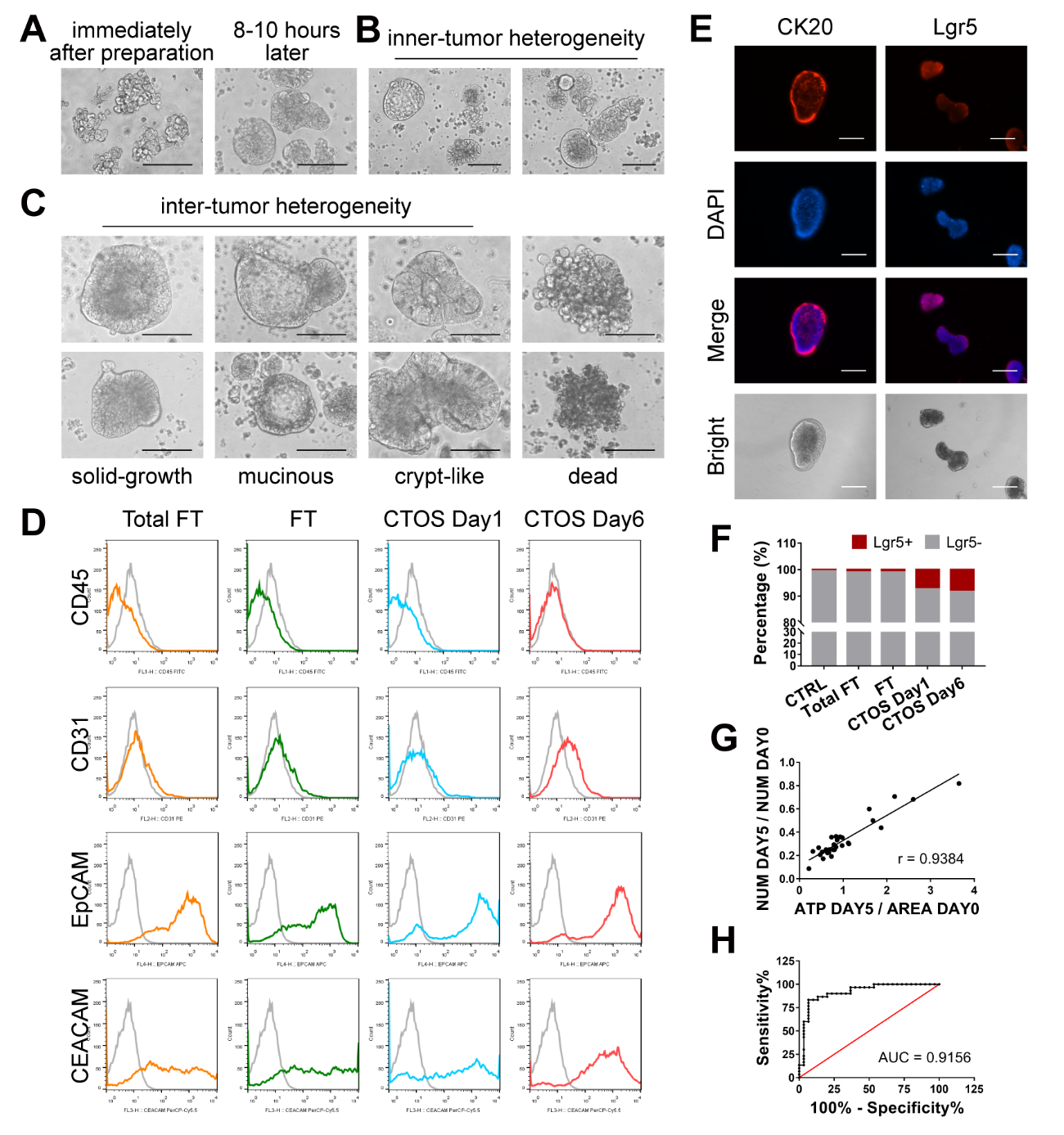


**Figure. S1 Phenotypes of colorectal CTOS. (A)** Microscopic images of CTOS immediately after preparation and 8–10 hours later. **(B-C)** Typical images of inner-tumor and inter-tumor heterogeneity in CRC CTOS. Morphological differences mainly manifested in size, shape, and appearance, including solid-growth, mucinous and crypt-like spheroids. **(D)** Flow cytometry analysis of total filtrate, filtrate (FT) and CTOS #14 cultured on day 1 and day 6, stained by epithelial markers CEACAM and EpCAM, immune cell marker CD45, and endothelial marker CD31. **(E)** Immunofluorescent staining of CK20 and Lgr5 using CTOS #22. **(F)** Flow cytometry analysis of total filtrate, FT and CTOS #22 cultured on day 1 and day 6, stained by Lgr5. **(G)** Correlation curve of organoid viability measured respectively by number ratio of spheroids on the fifth and first day, and ATP levels on the fifth day standardized by the original spheroid areas. r = 0.9384. **(H)** Receiver operating characteristic curve analysis of the predictive value of number ratio to evaluate organoid viability. AUC = 0.9156. Scale bar = 200 μm.

**
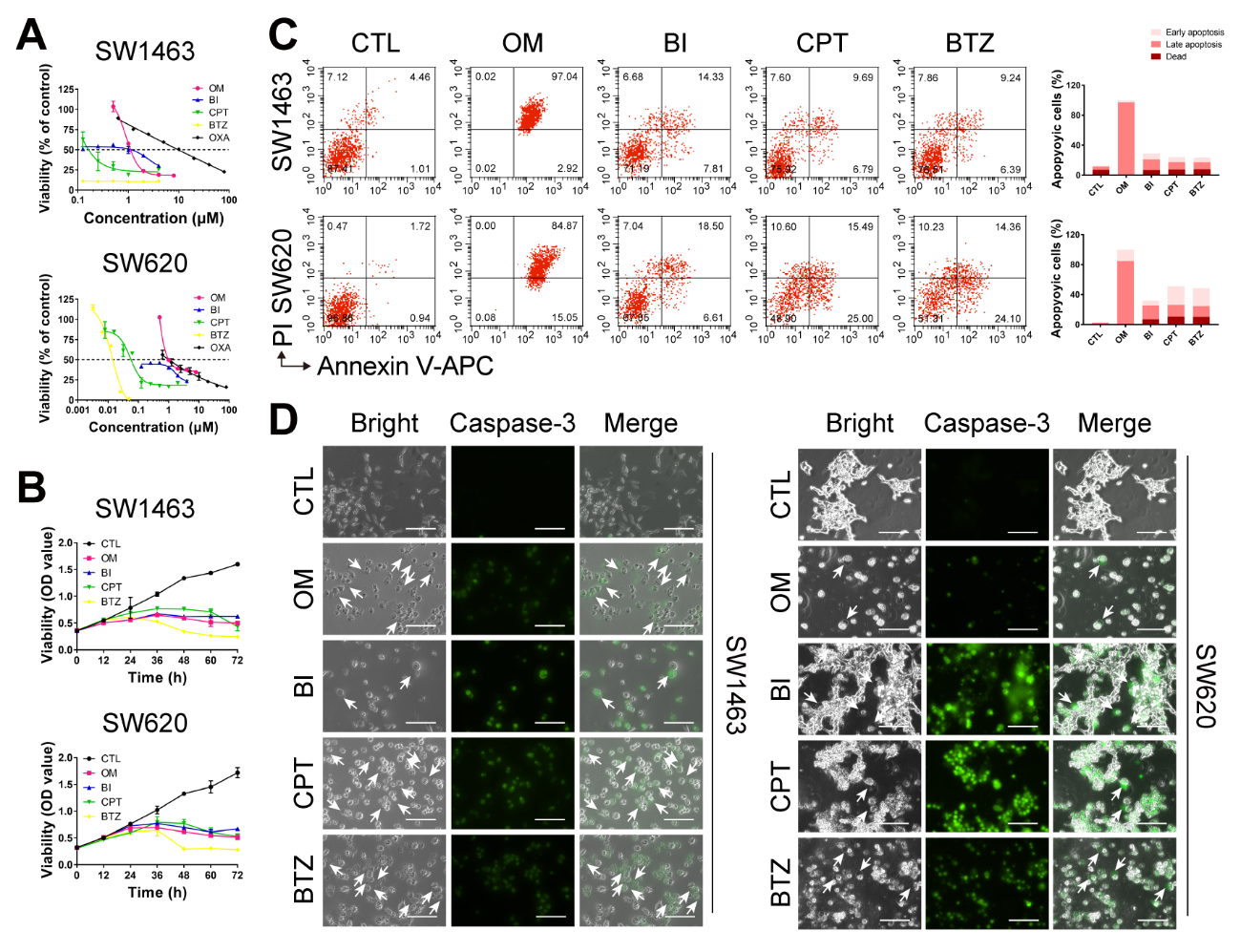
**

**Figure. S2 Small molecule inhibitors induce apoptosis in colorectal cancer cell lines.** **(A)** Half-maximal inhibitory concentration curves of four drug candidates in drug screen evaluated by CCK-8 assay. IC50 of each drug was lower than that of oxaliplatin (OXA) in SW1463 and SW620 cells. **(B)** Time-viability curve for treatment with OM, BI, CPT and BTZ over 72 hours after drug application. **(C)** Flow cytometry analysis of Annexin Ⅴ-APC and PI staining of apoptotic cells. SW1463 and SW620 cells were treated with OM, BI, CPT and BTZ for three days. **(D)** Representative images showing immunofluorescence staining of inhibitor-treated cells for three days showing the enzymatic activity of caspase-3. The white arrows point to balloon-like pyroptotic cells. Scale bar = 100 μm.

**
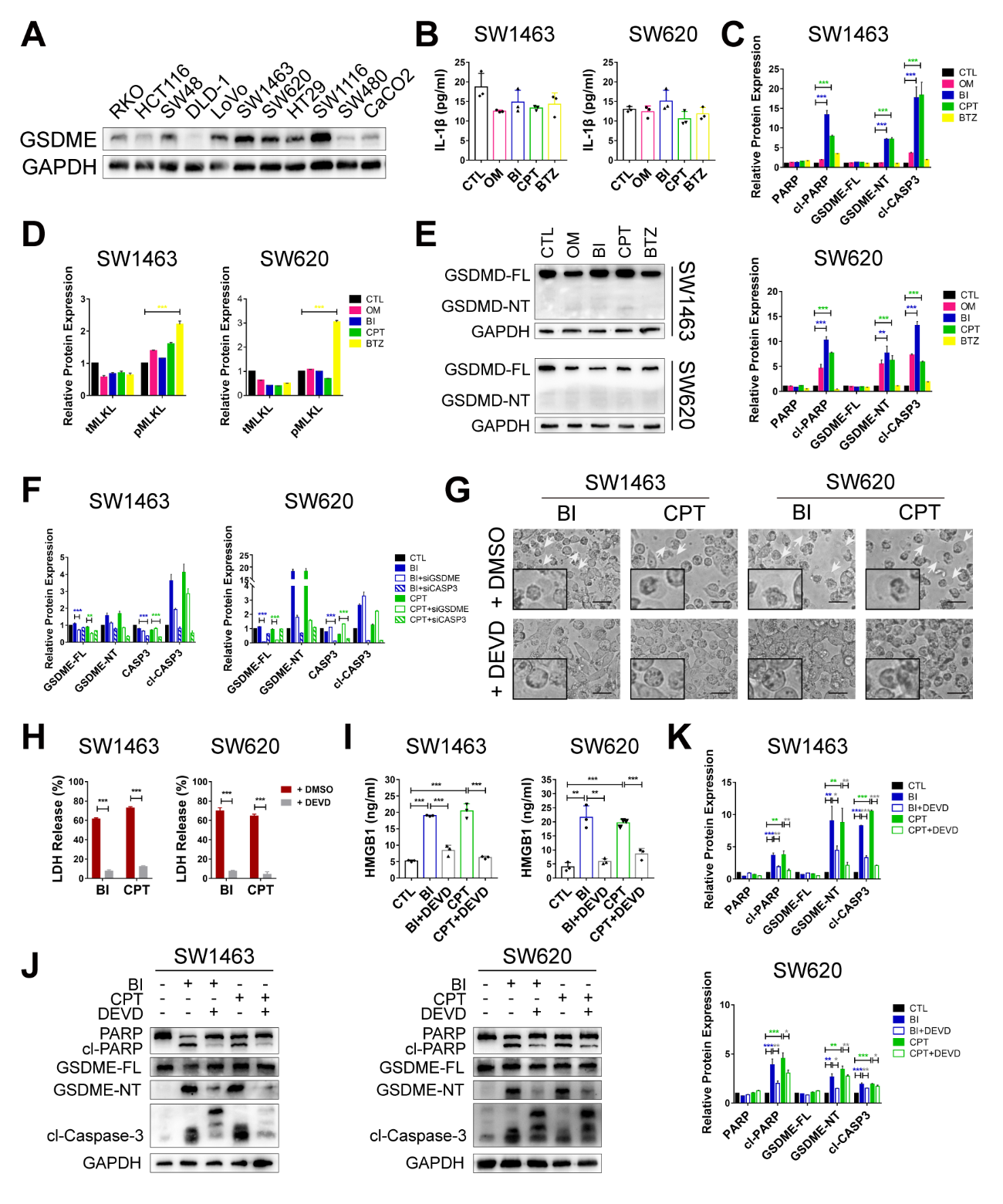
**

**Figure. S3 BI and CPT induce pyroptosis by caspase-3/GSDME pathway.** **(A)** Immunoblotting analysis of GSDME expression in colorectal cancer cell lines. **(B)** IL-1β release from SW1463 and SW620 cells treated with indicated inhibitors. Each column represents the mean value of three biological replicates, and error bars indicate SD. **(C,D,F)** Relative protein expression normalized by GAPDH. (C-D) related to Figure 3D and E. (F) related to Figure 3J. **(E)** Immunoblotting analysis of the indicated proteins extracted from inhibitor-treated cells. No cleavage of GSDMD was observed. **(G)** Bright-field microscopy images of indicated inhibitor-treated cells. Altered morphology was shown when co-treated with caspase-3 inhibitor DEVD. **(H)** LDH and **(I)** HMGB1 release of colorectal cancer cells treated with BI and CPT. The level of LDH decreased when adding DEVD. **(J-K)** Immunoblotting analysis of the indicated proteins extracted from inhibitor-treated cells and relative protein expression normalized by GAPDH. BI and CPT induced cleavage of PARP, GSDME and caspase-3, which was inhibited by DEVD. Scale bar = 200 μm.

**
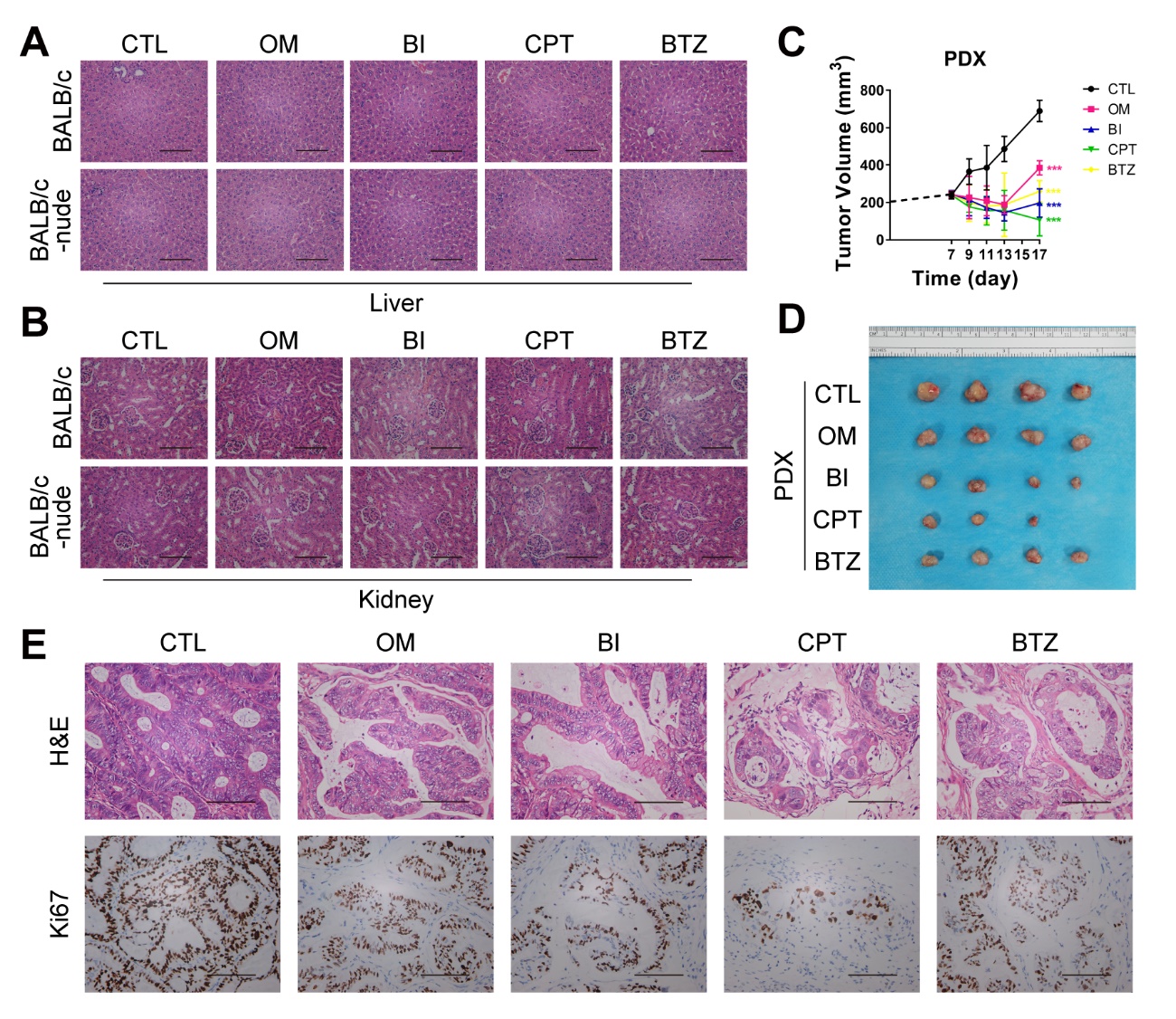
**

**Figure. S4 Histopathological analysis of the liver and kidney and the tumor inhibition effect in patient-derived xenograft model.** **(A-B)** H&E staining showed no damage to the liver and kidney when treated by DMSO, OM, BI, CPT and BTZ. **(C)** Tumor growth curves and **(D)** images of patient-derived xenograft (PDX) tumors with DMSO, OM, BI, CPT and BTZ treatment among BALB/c-nude mice. **(E)** H&E and Ki67 staining of drug-treated subcutaneous tumors in PDX model. Scale bar = 200μm.

**Supplementary Tables**

**Table. S1 Clinical information of the CRC patients CTOS originated.**

| Sample | Sex | Age [year] | Tumor location | Pathological stage | T stage | N stage | M stage | Tumor differentiation |
| --- | --- | --- | --- | --- | --- | --- | --- | --- |
| #1 | M^1^ | 81 | R^3^ | ⅢB | 3 | 1b | 0 | Mo^8^ |
| #2 | F^2^ | 74 | R | ⅡA | 3 | 0 | 0 | Mo |
| #3 | M | 67 | SC^4^ | Ⅰ | 2 | 0 | 0 | Mo |
| #4 | M | 56 | TC^5^ | Ⅰ | 2 | 0 | 0 | Mo |
| #5 | F | 65 | R | ⅢA | 2 | 1b | 0 | Mo |
| #6 | M | 62 | R | ⅡB | 4a | 0 | 0 | Mo |
| #7 | F | 63 | R | 0 | Tis | 0 | 0 | G^9^ |
| #8 | M | 63 | R | Ⅰ | 2 | 0 | 0 | Mo |
| #9 | M | 55 | R | ⅢC | 3 | 2b | 0 | Mo |
| #10 | F | 70 | SC | ⅢB | 3 | 1b | 0 | Mo |
| #11 | F | 72 | SC | Ⅳ | 3 | 1b | 1a | Mo |
| #12 | F | 75 | R | ⅡA | 3 | 0 | 0 | Mo |
| #13 | M | 54 | SC | ⅢB | 3 | 1b | 0 | Mo |
| #14 | M | 86 | AC^6^ | ⅡA | 3 | 0 | 0 | Mo |
| #15 | F | 76 | R | 0 | Tis | 0 | 0 | G |
| #16 | F | 67 | DC^7^ | ⅡA | 3 | 0 | 0 | Mo – P^10^ |
| #17 | M | 65 | AC | ⅡA | 3 | 0 | 0 | Mo |
| #18 | M | 46 | DC | ⅢC | 3 | 2b | 0 | Mo |
| #19 | F | 65 | AC | ⅢB | 3 | 1a | 0 | Mo - P |
| #20 | F | 85 | TC | ⅡA | 3 | 0 | 0 | Mo |
| #21 | M | 40 | SC | ⅡA | 3 | 0 | 0 | Mo - P |
| #22 | M | 47 | R | Ⅳ | 3 | 2a | 1a | Mo |
| PDX | M | 60 | TC | ⅡA | 3 | 0 | 0 | Mo |

^1^ M: male; ^2^ F: female; ^3^ R: rectum; ^4^ SC: sigmoid colon; ^5^ TC: transverse colon; ^6^ AC: ascending colon; ^7^ DC: descending colon; ^8^ Mo: moderate; ^9^ G: good; ^10^ P: poor

Table S1 (continued)

| Sample | MLH1 | MSH2 | MSH6 | PMS2 | EGFR | Her-2 | *EBER* | *KRAS* | *NRAS* | *PIK3CA* | *BRAF* |
| --- | --- | --- | --- | --- | --- | --- | --- | --- | --- | --- | --- |
| #1 | + | + | + | + | + | / | - | / | / | / | / |
| #2 | + | + | + | + | + | / | - | / | / | / | / |
| #3 | + | + | + | + | + | / | - | / | / | / | / |
| #4 | - | + | + | - | + | / | - | / | / | / | / |
| #5 | + | + | + | + | + | / | - | / | / | / | / |
| #6 | + | + | + | + | + | / | - | / | / | / | / |
| #7 | + | + | + | + | + | / | - | / | / | / | / |
| #8 | + | + | + | + | / | + | - | / | / | / | / |
| #9 | + | + | + | + | / | - | - | - | - | - | - |
| #10 | + | + | + | + | + | / | - | / | / | / | / |
| #11 | + | + | + | + | + | / | - | + | - | - | - |
| #12 | + | + | + | + | + | / | - | / | / | / | / |
| #13 | + | + | + | + | / | - | - | / | / | / | / |
| #14 | + | + | + | + | / | / | - | / | / | / | / |
| #15 | / | / | / | / | / | / | / | / | / | / | / |
| #16 | + | + | + | + | / | + | - | / | / | / | / |
| #17 | + | + | + | + | + | / | - | / | / | / | / |
| #18 | + | + | - | + | + | / | - | + | - | - | - |
| #19 | + | + | - | + | / | / | - | / | / | / | / |
| #20 | - | + | + | - | + | / | - | / | / | / | / |
| #21 | + | + | + | + | + | / | - | / | / | / | / |
| #22 | + | + | + | + | / | - | - | - | - | - | - |
| PDX | + | + | + | + | + | / | - | / | / | / | / |

**Table. S2 Compounds used in drug screen.**

| Drug | Target | Pathway^1^ | Action |
| --- | --- | --- | --- |
| Doramapimod | p38 MAPK | MAPK signaling | Inhibitor |
| JNK Inhibitor VIII | JNK | MAPK signaling | Inhibitor |
| Dabrafenib | B-Raf, C-Raf | MAPK signaling | Inhibitor |
| Vemurafenib | B-Raf | MAPK signaling | Inhibitor |
| Trametinib | MEK1, MEK2 | MAPK signaling | Inhibitor |
| LGK974 | PORCN | Wnt signaling | Inhibitor |
| Galunisertib | TGFβR1 | TGF-beta signaling | Inhibitor |
| Lenalidomide | TNF | TGF-beta signaling | Inhibitor |
| PAC1 | caspase-3 | Apoptosis | antagonist |
| Embelin | XIAP | Apoptosis | Inhibitor |
| Obatoclax Mesylate | Bcl-2 | Apoptosis | antagonist |
| Serdemetan | HDM2 | Cell cycle, p53 signaling | antagonist |
| Adavosertib | WEE1 | Cell cycle | inhibitor |
| GSK269962A | ROCK | TGF-beta signaling | inhibitor |
| AZD7762 | Chk1, Chk2 | Two-component system | inhibitor |
| EHT 1864 | Rac1, Rac1b, Rac2, Rac3 | MAPK signaling, Ras signaling | inhibitor |
| Alisertib | Aurora kinase A | Cell cycle | inhibitor |
| Palbociclib Isethionate | CDK4, CDK6 | Cell cycle | inhibitor |
| BI 2536 | Plk1 | Cell cycle | inhibitor |
| Sorafenib tosylate | Multi-kinase (B-Raf, c-Kit, PDGFRβ, VEGFR2 et al.) | MAPK signaling, mTOR signaling, Ras signaling, PI3K-Akt signaling | inhibitor |
| Tandutinib | FLT3, PDGFRβ | MAPK signaling, Ras signaling | antagonist |
| Regorafenib | Multi-kinase (Ret, C-Raf, VEGFR2, c- Kit et al.) | MAPK signaling | inhibitor |
| Motesanib | VEGFR, PDGFR, c- Kit | MAPK signaling, Ras signaling | inhibitor |
| Lapatinib Ditosylate | EGFR, ErbB2 | ErbB signaling, MAPK signaling, PI3K-Akt signaling | inhibitor |
| Imatinib Mesylate | c-Kit, PDGFR, ABL1 | MAPK signaling, Ras signaling, ErbB signaling | inhibitor |
| Nilotinib | Bcr-Abl | Ras signaling, ErbB signaling | inhibitor |
| Saracatinib | c-Src, Lck, Fyn, Lyn, Blk, Fgr, c-Yes | NF-kappa B signaling, Chemokine signaling | inhibitor |
| Crizotinib | c-Met, ALK | MAPK signaling, Ras signaling, PI3K-Akt signaling | inhibitor |
| Afatinib | EGFR, HER2, HER4 | MAPK signaling, ErbB signaling, PI3K-Akt signaling | inhibitor |
| Brivanib Alaninate | VEGFR2, FGFR1 | MAPK signaling, Ras signaling | inhibitor |
| Ruxolitinib phosphate | JAK1, JAK2 | JAK-STAT signaling, PI3K-Akt signaling | inhibitor |
| Acadesine | AMPK | AMPK signaling, mTOR signaling, PI3K-Akt signaling | antagonist |
| BX795 | PDK1 | PPAR signaling, mTOR signaling, PI3K-Akt signaling | inhibitor |
| PF4708671 | p70S6K1 | ErbB signaling, mTOR signaling, PI3K-Akt signaling | inhibitor |
| Everolimus | mTOR | mTOR signaling, PI3K-Akt signaling | inhibitor |
| Alpelisib | PI3Kα | PI3K-Akt signaling, mTOR signaling | inhibitor |
| CHIR99021 | GSK-3α, GSK-3β | Chemokine signaling | inhibitor |
| (S)-crizotinib | MTH1 | DNA Repair^2^ | inhibitor |
| BIBR 1532 | TEP1 | DNA replication^2^ | inhibitor |
| (S)-(+)-Camptothecin | Top1 | DNA replication^2^ | inhibitor |
| Gemcitabine HCl | DNA synthesis^3^ | DNA synthesis^3^ | inhibitor |
| CP466722 | ATM | NF-kappa B signaling, p53 signaling, Cell cycle | inhibitor |
| XAV939 | TNKS1, TNKS2 | DNA replication^2^ | inhibitor |
| Enzastaurin | PKCβ | MAPK signaling, Ras signaling, NF-kappa B signaling | inhibitor |
| Docetaxel | microtubule^3^ | microtubule disassembly^3^ | inhibitor |
| MK-2206 dihydrochloride | Akt1, Akt2, Akt3 | PI3K-Akt signaling, mTOR signaling | inhibitor |
| Tanespimycin | HSP90 | PI3K-Akt signaling, NOD-like receptor signaling | inhibitor |
| Vorinostat | HDAC | Cell cycle, Notch signaling | inhibitor |
| Olaparib | PARP1, PARP2 | Apoptosis, NF-kappa B signaling | inhibitor |
| UNC0642 | G9a, GLP1 | Metabolic pathways, cAMP signaling | inhibitor |
| Bortezomib | PSMA, PSMB | Proteasome | inhibitor |
| Sonidegib | Smo | Hedgehog signaling | antagonist |
| Fulvestrant | ER | Estrogen signaling | antagonist |
| FK866 | NAMPT | Metabolic pathways | inhibitor |
| Methotrexate | DHFR | Metabolic pathways | inhibitor |
| BMS345541 | IKK1, IKK2 | NF-kappa B signaling, mTOR signaling, PI3K-Akt signaling | inhibitor |
| Tepotinib | c-Met | MAPK signaling, Ras signaling, PI3K-Akt signaling | inhibitor |
| Linsitinib | IGF1R | MAPK signaling, Ras signaling, HIF-1 signaling | inhibitor |
| Vinblastine sulfate | nAChR | ERK signaling, JAK-STAT signaling | inhibitor |
| Avagacestat | Aph1, Pen2 | Notch signaling | inhibitor |

^1^ The terms are based on KEGG Pathway (https://www.kegg.jp/). Only three main pathways are shown. ^2^ These terms refer to the function of the target proteins without specific pathway term in KEGG. ^3^ It refers to the main function of the drug which has no specific target.

**Table. S3 Primers used in Sanger sequencing**

|  |  | Primer sequences |
| --- | --- | --- |
| *KRAS* exon 2 | forward | GGTACTGGTGGAGTATTTGATAG |
|  | reverse | ATAACTTGAAACCCAAGGTACA |
|  | sequence | CATGAAAATGGTCAGAGAAACCT |
| *TP53* exon 7 | forward | AGGTCTCCCCAAGGCGCACT |
|  | reverse | AAAGAAAACTGAGTGGGAGCAGT |
|  | sequence | AGTAGTATGGAAGAAATCGGT |
| *TP53* exon 8 | forward | AAGGGTGGTTGGGAGTAGATG |
|  | reverse | AATATTCTCCATCCAGTGGTTTC |
|  | sequence | CTGGTGTTGTTGGGCAGTGCT |
| *BRAF* | forward | GCTTGCTCTGATAGGAAAATGAG |
|  | reverse | GTAACTCAGCAGCATCTCAGG |
| *PIK3CA* | forward | GCTTTTTCTGTAAATCATCTGTG |
|  | reverse | CTGAGATCAGCCAAATTCAGT |

**Table. S4 Typical genomic feature of CTOS #1-10 and corresponding tumors.**

| Sample | | *KRAS* | *BRAF* | *TP53* | *PIK3CA* |
| --- | --- | --- | --- | --- | --- |
| #1 | Tissue | null | null | null | null |
|  | CTOS | null | null | null | null |
| #2 | Tissue | null | null | null | null |
|  | CTOS | null | null | null | null |
| #3 | Tissue | null | null | S241T | null |
|  | CTOS | null | null | S241T | null |
| #4 | Tissue | null | null | G245S | null |
|  | CTOS | null | null | G245S | null |
| #5 | Tissue | null | null | null | null |
|  | CTOS | null | null | null | null |
| #6 | Tissue | G12V | null | R273C | null |
|  | CTOS | G12V | null | R273C | null |
| #7 | Tissue | null | null | null | null |
|  | CTOS | null | null | null | null |
| #8 | Tissue | G12D | null | null | null |
|  | CTOS | G12D | null | null | null |
| #9 | Tissue | null | null | R248Q | null |
|  | CTOS | null | null | R248Q | null |
| #10 | Tissue | G12D | null | null | null |
|  | CTOS | G12D | null | null | null |

**Table. S5 The data related to Figure 2B.**

| CTOS | #18 | #19 | #20 | #21 |
| --- | --- | --- | --- | --- |
| Doramapimod | 1.03 | 0.83 | 0.77 | 0.81 |
| JNK Inhibitor VIII | 1.32 | 0.57 | 0.80 | 0.54 |
| Dabrafenib | 0.74 | 0.85 | 0.78 | 0.84 |
| Vemurafenib | 0.88 | 0.77 | 0.76 | 0.97 |
| Trametinib | 0.46 | 0.10 | 0.77 | 0.00 |
| LGK974 | 1.26 | 0.78 | 0.74 | 1.17 |
| Galunisertib | 0.86 | 0.61 | 0.91 | 0.84 |
| Lenalidomide | 1.11 | 0.63 | 0.72 | 0.90 |
| PAC1 | 0.96 | 0.39 | 0.59 | 0.71 |
| Embelin | 1.31 | 0.81 | 0.60 | 0.79 |
| Obatoclax Mesylate | 0.00 | 0.00 | 0.00 | 0.00 |
| Serdemetan | 0.39 | 0.36 | 0.17 | 0.77 |
| Adavosertib | 0.99 | 0.67 | 0.45 | 0.88 |
| GSK269962A | 0.97 | 0.62 | 0.55 | 0.94 |
| AZD7762 | 0.33 | 0.25 | 0.25 | 0.60 |
| EHT 1864 | 0.95 | 0.65 | 0.71 | 1.08 |
| Alisertib | 1.07 | 0.66 | 0.61 | 0.73 |
| Palbociclib Isethionate | 0.73 | 0.55 | 0.68 | 1.16 |
| BI 2536 | 0.00 | 0.00 | 0.00 | 0.00 |
| Sorafenib tosylate | 0.90 | 0.66 | 0.81 | 0.96 |
| Tandutinib | 0.91 | 0.57 | 0.48 | 1.14 |
| Regorafenib | 0.84 | 0.57 | 0.70 | 1.42 |
| Motesanib | 0.91 | 0.40 | 0.64 | 1.09 |
| Lapatinib Ditosylate | 0.90 | 0.42 | 0.73 | 1.00 |
| Imatinib Mesylate | 0.90 | 0.68 | 0.59 | 1.20 |
| Nilotinib | 0.84 | 0.62 | 0.50 | 1.04 |
| Saracatinib | 1.07 | 0.58 | 0.68 | 1.00 |
| Crizotinib | 0.91 | 0.74 | 0.53 | 0.82 |
| Afatinib | 0.79 | 0.73 | 0.79 | 0.78 |
| Brivanib Alaninate | 0.86 | 0.77 | 0.53 | 0.69 |
| Ruxolitinib phosphate | 0.84 | 0.59 | 0.44 | 0.97 |
| AICAR (Acadesine) | 0.89 | 0.68 | 0.56 | 1.00 |
| BX795 | 0.84 | 0.26 | 0.03 | 0.86 |
| PF4708671 | 0.97 | 0.49 | 0.62 | 1.05 |
| Everolimus | 0.91 | 0.30 | 0.48 | 0.91 |
| Alpelisib | 1.01 | 0.36 | 0.66 | 0.85 |
| CHIR99021 | 0.98 | 0.59 | 0.63 | 0.75 |
| (S)-crizotinib | 0.43 | 0.21 | 0.42 | 0.68 |
| BIBR 1532 | 1.00 | 0.57 | 0.52 | 0.85 |
| (S)-(+)-Camptothecin | 0.00 | 0.30 | 0.00 | 0.00 |
| Gemcitabine HCl | 0.82 | 0.23 | 0.50 | 0.73 |
| CP466722 | 0.91 | 0.27 | 0.59 | 1.00 |
| XAV939 | 1.05 | 0.46 | 0.55 | 0.95 |
| Enzastaurin | 0.76 | 0.51 | 0.38 | 1.06 |
| Docetaxel | 0.77 | 0.35 | 0.42 | 0.59 |
| MK-2206 dihydrochloride | 0.81 | 0.23 | 0.48 | 1.11 |
| Tanespimycin | 0.87 | 0.28 | 0.68 | 0.32 |
| Vorinostat | 0.46 | 0.45 | 0.38 | 0.38 |
| Olaparib | 0.73 | 0.45 | 0.66 | 0.91 |
| UNC0642 | 0.75 | 0.45 | 0.44 | 1.13 |
| Bortezomib | 0.00 | 0.00 | 0.00 | 0.00 |
| Sonidegib | 0.12 | 0.34 | 0.53 | 1.14 |
| Fulvestrant | 0.73 | 0.37 | 0.61 | 0.91 |
| FK866 | 0.80 | 0.20 | 0.46 | 0.49 |
| Methotrexate | 0.75 | 0.42 | 0.57 | 0.91 |
| BMS345541 | 0.10 | 0.12 | 0.00 | 0.29 |
| Tepotinib | 0.64 | 0.06 | 0.29 | 0.30 |
| OSI-906; Linsitinib | 0.90 | 0.34 | 0.54 | 0.79 |
| Vinblastine sulfate | 0.52 | 0.37 | 0.24 | 0.37 |
| Avagacestat | 0.92 | 0.49 | 0.57 | 0.78 |
